# Medical care costs of cancer in the last year of life using national health insurance data in Korea

**DOI:** 10.1371/journal.pone.0197891

**Published:** 2018-06-07

**Authors:** Mihai Park, Inmyung Song

**Affiliations:** 1 Pharmaceutical Benefits Listing Division, Health Insurance Review and Assessment Service, Seoul, Korea; 2 School of Pharmacy, Sungkyunkwan University, Suwon, Korea; Brown University, UNITED STATES

## Abstract

**Background:**

Medical care of cancer patients at the end-of-life is costly. This study aims to describe the monthly trends of EOL medical care, drug therapy, and chemotherapy costs per patient with cancer in the last year of life in the inpatients vs. outpatient setting for the 13 most prevalent cancers in Korea.

**Methods:**

Using the Health Insurance Review and Assessment Service (HIRA) database, we identified the patients who had been treated for the primary diagnoses of one of the 13 most prevalent cancers in Korea and died between January 1, 2013 and December 31, 2015. We calculated the mean monthly costs of medical care, drug therapy, and chemotherapy per patient in the last year of life by cancer site and patient setting (inpatient vs. outpatient).

**Results:**

For most cancers, the monthly inpatient costs per patient remain stable or increased gradually from 12 months to 3 months prior to death and then increased steeply from 2 months prior to death. The mean monthly inpatient costs per patient were highest for acute myeloid leukemia (AML) throughout the last year of life; all solid tumors had similar trends of monthly inpatient costs. The mean monthly inpatient costs for AML increased from $5,465 (SD, $5,248) in 12 months prior to death to $15,033 (SD, $11,864) in the last month. The monthly outpatient costs per patient showed similar, gradually decreasing trends for most cancers. The mean outpatient costs were highest for kidney cancer; the costs sharply decreased from $954 (SD, $1,346) in 12 months prior to death to $424 (SD, $736) in the last month. The proportion of inpatients receiving chemotherapy in the last month of life was highest for AML (77%), followed by liver cancer (67%) and breast cancer (56%).

**Conclusion:**

The monthly inpatient medical care costs per patient with cancer increased as the patient approached death, while the monthly outpatient costs decreased. A considerable proportion of inpatient received chemotherapy in the last month of life. Efforts are needed to optimize EOL care for cancer patients.

## Introduction

Cancer is a leading cause of death in Korea; in 2016, 27.8% of all deaths in Korea were from cancer [1,2]. Due to rapid aging of the population and changes in lifestyle, the number of cancer deaths has nearly tripled from 1983 to 2012 [3]. Consequently, the economic burden of cancer in Korea has nearly doubled over a 10-year period to approximately US$ 20 billion in 2010 [4,5]. Korea has a mandatory health insurance system which covers approximately 98% of its entire inhabitants and most medical procedures are reimbursed on a fee-for-service basis [6]. In this context, cancer-related medical expenditures have become a burden on the national health insurance budget, accounting for 9% of total spending in 2016 [7]. End-of-life (EOL) care, which consumed 8.3% of the budget in 2008, is likely to put an increasing onus on the insurance system [8].

Studies in other countries show that the amount of health care resources used increases as the patient approaches death [9,10]. For example, one fourth of all spending to care for Medicare beneficiaries in the United States occurred in the last year of life [9]. Approximately 10% of public health expenditures in Ontario, Canada were spent on medical care in the last year of life and EOL care spending rose sharply in the last three months [10]. The costs of EOL care for cancer patients can be particularly costly. The costs of EOL care were $10,000 more for Medicaid beneficiaries with cancer than for those without cancer in the United States [11]. In the Netherlands, the costs of EOL care were highest for cancer among major causes of death [12]. Despite differences across countries, the challenges of controlling EOL care costs for cancer are similar.

Examining the trends of monthly EOL care costs among cancer decedents can illuminate the increasingly high economic burden of cancer even within the last year of life. A study in Korea estimated that the costs of cancer care steadily increased until 2 months prior to death and that 69.5% of total medical care costs for cancer in the last year of life were spent in the last six months [13]. Another study in Korea showed that 50.4% of total medical care costs for cancer in the last year of life incurred in the last three months and monthly costs were highest in the last month, having doubled from the previous month [14]. In Taiwan, one third of EOL care costs for cancer incurred in the last month of life [15].

Terminal cancer patients were treated with multiple drugs for the management of the symptoms of cancer and comorbid conditions; some even received chemotherapy near the end of life [16,17]. A U.S. study found that 29.5% of older patients with Medicare received chemotherapy in the last year of life [17]. A greater proportion (50.3%) of cancer patients at a teaching hospital in Korea received chemotherapy during the last 2 months of life [16]. However, chemotherapy in the last week of life was associated with worse EOL outcomes and cost increases [18]. Little is known about the trends in medical care, drug therapy, and chemotherapy costs among patients with terminal cancer in Korea; it is imperative to understand the cost components and the stage of cancer care that contribute most to cost burden among cancer decedents. Therefore, based on the health insurance claims data that provide nationally representative cost information, this study aims to describe the trends of the mean monthly costs of medical care, drug therapy, and chemotherapy per patient with cancer in the last year of life in the inpatient vs. outpatient setting by cancer site among cancer decedents from the 13 most prevalent cancers in Korea.

## Materials and methods

### Database

We used the Health Insurance Review and Assessment Service (HIRA) database, which contains national health insurance claims data in Korea. The HIRA database includes cost information on all medical services and prescription drugs that are reimbursed by the health insurance authority. We identified the patients who had been treated for the primary diagnoses of one of the 13 most prevalent cancers in Korea and died between January 1, 2013 and December 31, 2015 by linking HIRA claims data with the mortality dates derived from the Statistic Korea. Personal identification numbers in the HIRA database were anonymized before providing to researchers. Diagnoses were coded according to the International Classification of Disease-10^th^ revision (ICD-10). The 13 cancers were stomach cancer (ICD-10 code, C16), liver cancer (C22), lung cancer (C34), breast cancer (C50), colorectal cancer (C18, C19, C20), kidney cancer (C64, C65), prostate cancer (C61), acute myeloid leukemia (AML; C920, C925, C926, C927, C928, C929, C930, C942), non-Hodgkin’s lymphoma (C85), cervical cancer (C53), ovarian cancer (C56), pancreatic cancer (C25), and thyroid cancer (C73).

### Monthly medical care costs per patient

We extracted claims data for the identified decedents for 360 days before death. We calculated the mean number of inpatient and outpatient days as well as the monthly medical care costs per patient by cancer site and patient setting (inpatient vs. outpatient). Monthly costs were calculated on the basis of 30 days per month by equally dividing 360 days into 12 months. Outpatient costs were determined by summing claimed amounts on all services provided for the decedents in the outpatient setting each month. Inpatient costs were assumed to be equally spread over the duration of hospitalization. In other words, total costs incurred during a period of hospitalization that lasted more than a day were divided by the number of inpatient days based on the assumption that costs per inpatient day were the same throughout the period. The resulting inpatient costs per day were multiplied by the number of inpatient days in a given month to produce the monthly inpatient costs. For example, if a patient was hospitalized on day 28 of month 11 and then discharged on day 2 of month 12, we counted 2 days in month 11 and 2 days in month 12. Costs in Korean won were converted to US$ using the conversion rate of 1,100 won/US$.

### Monthly costs of drug therapy and chemotherapy per patient

We calculated drug therapy costs as a proportion of total medical care costs, the proportion of patients receiving chemotherapy, and the ratio of mean drug therapy costs to mean chemotherapy costs in the last month of life by cancer site and patient setting. We also calculated the monthly costs of drug therapy and chemotherapy per patient in the last year of life. The mean drug therapy costs were defined as the mean of drug therapy costs per patient in all patients who were treated with prescription drugs each month. Only a portion of all cancer patients who were on drug therapy received chemotherapy. The mean chemotherapy costs were defined as the mean of chemotherapy costs per patient in all patients who were treated with chemotherapy each month. As a result, the mean chemotherapy costs per patient may be greater than the mean drug therapy costs per patient.

The mean and standard deviation (SD) were calculated for all continuous variables. All analyses were performed by using SAS-EG version 4.3 (Cary, NC, USA). The approval of the Institutional Review Board for this study with the general population was waived under Article 16 of the Rule of the Bioethics and Safety Act in Korea [19].

## Results

We included a total of 134,633 males and 75,039 females in the analysis (Table 1). The most common cancer was lung cancer for males (n = 39,142) and colorectal cancer for females (n = 13,883). The mean age ranged from 61 to 77 years for males and from 58 to 72 years for females depending on cancer site. For all cancers, the mean number of inpatient days per patient increased as the patient approached death; the number of inpatient days nearly doubled in the last month as compared to 12 months prior to death (Table 2). In contrast, the number of outpatient days remained stable.

**Table 1:** Study subjects.

Cancer site	Males (n = 134,633)			Females (n = 75,039)		
	No.	Mean age	SD	No.	Mean age	SD
Acute myeloid leukemia	1,674 (1.2%)	61	17.9	1,172 (1.6%)	61	18.3
Liver	29,327 (21.8%)	65	11.9	10,795 (14.4%)	71	12.1
Stomach	20,826 (15.5%)	69	12.2	9,888 (13.2%)	70	15.8
Breast	85 (0.1%)	71	12.0	7,482 (10.0%)	58	13.4
Prostate	8,304 (6.2%)	77	8.3	ㅡ	ㅡ	ㅡ
Non-Hodgkin’s lymphoma	1,738 (1.3%)	66	14.8	1,161 (1.5%)	69	14.5
Thyroid	832 (0.6%)	67	12.5	2,069 (2.8%)	68	13.2
Cervical	ㅡ	ㅡ	ㅡ	3,176 (4.2%)	63	15.8
Pancreatic	9,006 (6.7%)	68	11.2	7,288 (9.7%)	72	11.3
Lung	39,142 (29.1%)	71	9.9	13,480 (18.0%)	71	12.6
Ovarian	ㅡ	ㅡ	ㅡ	3,503 (4.7%)	62	14.1
Colorectal	20,794 (15.4%)	69	11.9	13,883 (18.5%)	72	13.3
Kidney	2,905 (2.2%)	68	12.3	1,142 (1.5%)	71	13.8

**Table 2:** Number of inpatient and outpatient days per patient with cancer by month in the last year of life.

Cancer site	Inpatient days												Outpatient days											
	12	11	10	9	8	7	6	5	4	3	2	1	12	11	10	9	8	7	6	5	4	3	2	1
Acute myeloid leukemia.	12	16	16	16	16	16	16	17	17	18	18	20	3	3	3	3	3	3	3	3	3	3	3	3
Liver	8	9	10	10	10	10	11	11	12	13	15	18	2	2	2	2	2	3	3	3	3	3	3	2
Stomach	9	11	11	11	11	11	12	12	13	14	16	20	2	2	2	2	2	2	2	3	3	3	3	2
Breast	9	11	12	12	12	12	13	13	14	15	16	19	3	3	3	3	3	3	3	3	3	3	3	3
Prostate	11	15	16	15	16	16	16	17	16	17	17	18	2	2	2	2	2	2	2	2	2	2	2	2
Non -Hodgkin’s lymphoma	9	12	12	12	13	14	14	14	15	16	16	18	3	2	2	2	2	2	3	3	3	2	2	2
Thyroid	10	16	16	16	16	18	17	18	17	17	18	19	2	2	2	2	2	2	2	2	2	2	2	2
Cervical	9	11	12	12	13	13	13	14	15	16	18	21	4	4	4	4	4	4	4	3	3	3	3	2
Pancreatic	9	11	11	11	11	11	11	12	13	14	16	20	4	3	3	3	3	3	3	3	3	3	3	3
Ovarian	9	11	11	11	12	12	12	13	14	15	18	21	3	3	3	3	3	3	3	3	3	3	3	3
Lung	8	10	10	11	11	11	11	12	13	14	16	19	3	3	3	3	3	3	3	3	3	3	3	2
Colorectal	9	12	12	12	13	13	14	14	15	16	18	20	3	3	3	3	3	3	3	3	3	3	3	2
Kidney	9	12	12	12	12	13	14	14	14	15	16	19	2	2	2	3	3	3	3	3	3	3	3	2

For most cancers, the monthly inpatient costs per patient remain stable or increased gradually from 12 months to 3 months prior to death and then increased steeply from 2 months prior to death (Table 3, S1 Table). The mean monthly inpatient costs per patient were highest for AML throughout the last year of life; all solid tumors had similar trends of monthly inpatient costs. The mean monthly inpatient costs for AML increased from $5,465 (SD, $5,248) in 12 months prior to death to $15,033 (SD, $11,864) in the last month. In contrast, the mean monthly outpatient costs showed similar, gradually downward trends for most cancers. The mean outpatient costs were highest for kidney cancer; the costs sharply decreased from $954 (SD, $1,346) in 12 months prior to death to $424 (SD, $736) in the last month. The mean outpatient costs for most of the other solid tumors remained stable and then slightly decreased as the patients approached death.

**Table 3:** Monthly inpatient and outpatient costs per patient with cancer in the last year of life.

Cancer site	Month before death	12		6		3		2		1	
		Mean costs ($)	SD	Mean costs ($)	SD	Mean costs ($)	SD	Mean costs ($)	SD	Mean costs ($)	SD
Acute myeloid leukemia	inpatient	5,465	5,248	8,257	7,013	9,684	8,074	10,972	9,487	15,033	11,864
	outpatient	471	689	553	898	517	837	521	831	415	661
Stomach	inpatient	1,771	1,692	2,130	1,900	2,504	2,244	2,872	2,629	3,791	3,691
	outpatient	336	459	302	428	295	422	282	406	241	348
Liver	inpatient	2,472	3,546	2,656	2,961	2,863	3,509	3,156	3,799	4,303	5,252
	outpatient	489	770	506	828	460	763	394	646	286	455
Lung	inpatient	1,869	1,807	2,275	2,088	2,629	2,455	2,954	2,742	3,889	3,806
	outpatient	598	890	534	856	485	801	437	717	322	530
Breast	inpatient	1,571	1,498	2,043	1,997	2,547	2,217	2,897	2,494	3,953	3,596
	outpatient	600	816	560	783	520	765	437	652	341	518
Colorectal	inpatient	1,882	1,735	2,230	2,087	2,553	2,633	2,837	2,916	3,626	4,060
	outpatient	359	568	339	554	323	523	302	472	247	367
Kidney	inpatient	1,922	2,221	2,314	2,331	2,778	2,648	2,940	2,734	4,086	4,597
	outpatient	954	1,346	831	1,248	700	1,092	583	1,031	424	736
Prostate	inpatient	1,288	1,173	1,822	1,495	2,129	2,009	2,356	2,458	3,008	3,478
	outpatient	357	769	355	670	373	705	351	676	294	520
Non-Hodgkin’s lymphoma	inpatient	2,209	2,713	3,560	4,069	4,232	5,201	4,514	5,147	6,630	8,213
	outpatient	356	594	372	695	336	665	340	716	292	709
Cervical	inpatient	1,760	1,490	2,402	2,236	3,269	3,153	3,623	3,138	4,294	3,851
	outpatient	596	1,013	482	871	363	681	317	559	259	449
Ovarian	inpatient	1,746	1,446	2,315	2,087	3,056	2,900	3,590	3,218	4,603	4,344
	outpatient	298	414	306	465	286	529	283	469	247	366
Pancreas	inpatient	2,306	2,369	2,380	2,273	2,670	2,297	2,979	2,474	3,860	3,211
	outpatient	583	809	505	711	405	591	351	492	277	393
Thyroid	inpatient	1,329	1,261	2,217	2,219	2,427	2,361	2,914	2,963	3,494	3,973
	outpatient	282	718	363	840	352	838	334	820	248	470

Costs in Korean won were converted to US$ using the conversion rate of 1,100 won/US$.

Among the patients who incurred inpatient costs in the last month of life, approximately 20% of the inpatient medical costs were spent on drug therapy for most cancers; however, the proportion was 30% for AML (Table 4). The proportion of inpatients receiving chemotherapy in the last month was highest for AML (77%), followed by liver cancer (67%) and breast cancer (56%). The ratio of the mean chemotherapy cost to mean drug therapy cost was highest for prostate cancer and non-Hodgkin’s lymphoma (both 23%), followed by kidney cancer (22%). In the outpatient setting, over 30% of total medical care costs in the last month were spent on drug therapy for most cancers; the percentage was highest for kidney cancer (87%), followed by prostate cancer (71%) and pancreatic cancer (64%). The proportion of outpatients receiving chemotherapy was highest for prostate cancer (33%), followed by AML (29%), lung cancer and pancreatic cancers (28%), and kidney cancer (27%); the proportion was lowest for thyroid cancer (7%). The ratio of the mean chemotherapy costs to the mean drug therapy costs in the outpatient setting was highest for thyroid cancer (1.98), followed by kidney cancer (1.96).

**Table 4:** Drug therapy costs, proportion of patients receiving chemotherapy, and chemotherapy to drug therapy cost ratio in the last month of life.

Cancer site	Drug therapy costs (proportion of total medical care costs)		Proportion of patients receiving chemotherapy		Mean chemotherapy costs/mean drug therapy costs	
	Inpatient	Outpatient	Inpatient	Outpatient	Inpatient	Outpatient
Acute myeloid leukemia	30%	47%	77%	29%	12%	88%
Stomach	25%	55%	42%	16%	13%	85%
Liver	22%	58%	67%	22%	8%	119%
Lung	24%	62%	51%	28%	18%	117%
Breast	22%	53%	56%	24%	18%	127%
Colorectal	25%	55%	31%	14%	15%	70%
Kidney	23%	87%	51%	27%	22%	196%
Prostate	22%	71%	39%	33%	23%	122%
Non-Hodgkin's lymphoma	24%	37%	44%	14%	23%	51%
Cervical	24%	49%	43%	13%	11%	50%
Ovarian	24%	55%	46%	9%	20%	119%
Pancreatic	25%	64%	55%	28%	8%	79%
Thyroid	21%	52%	31%	7%	9%	198%

**Fig 1:**
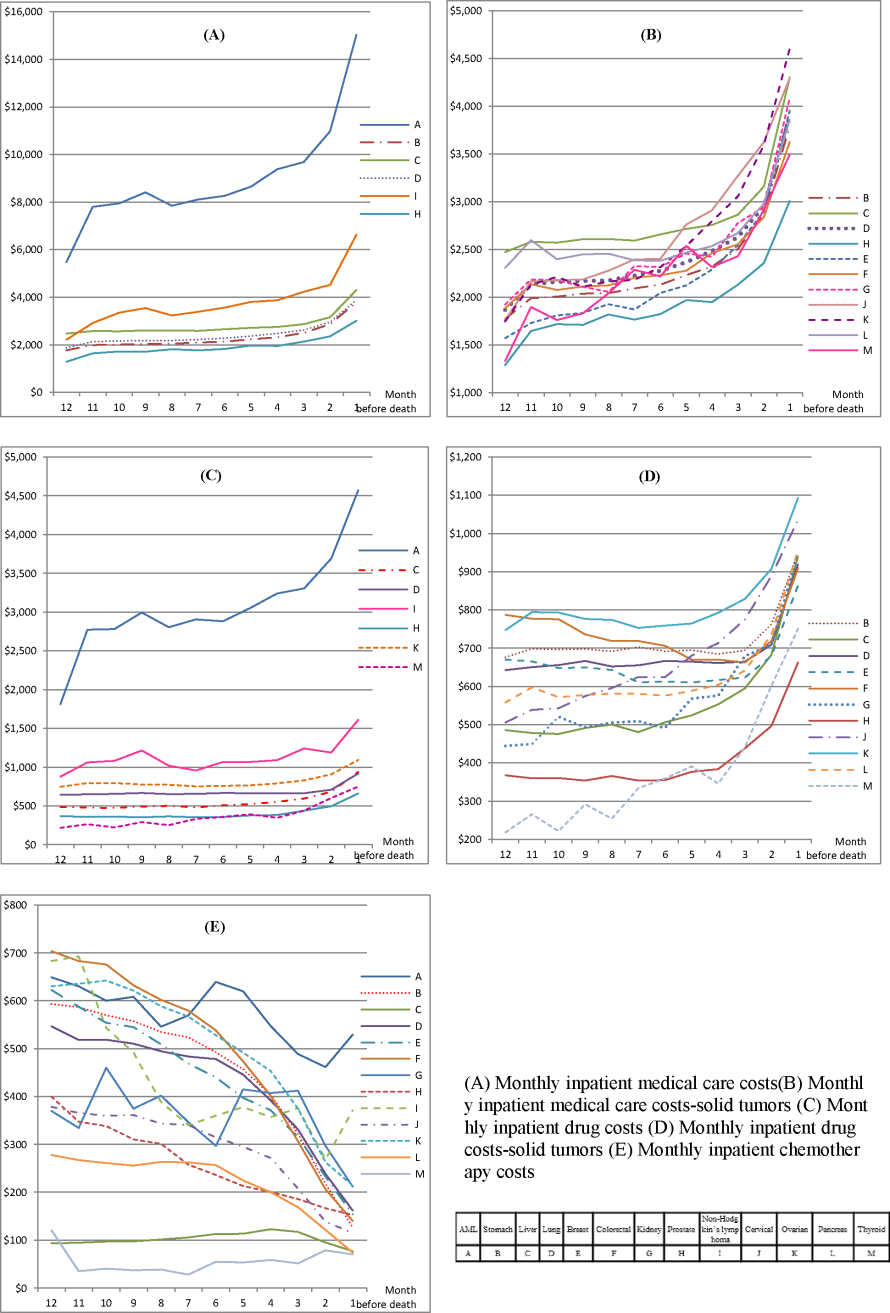
Mean monthly costs of inpatient medical care, drug therapy, chemotherapy.

**Fig 2:**
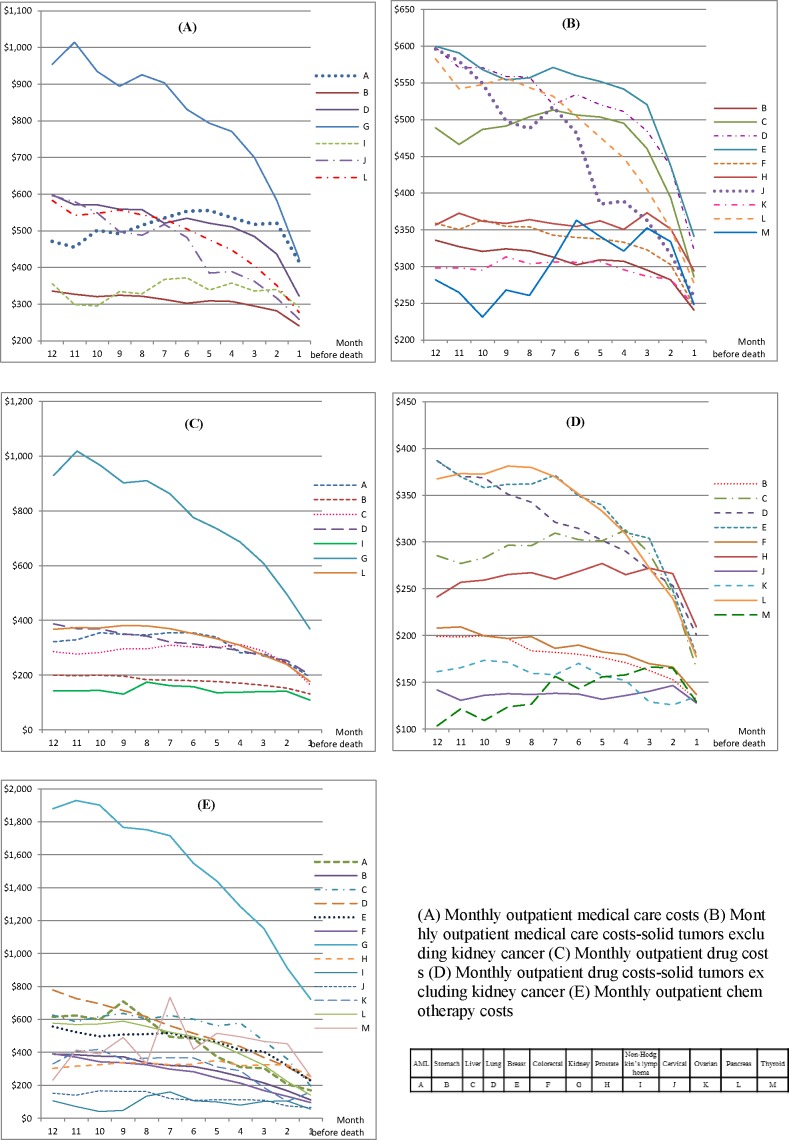
Mean monthly costs of outpatient medical care, drug therapy, and chemotherapy.

For inpatients, the mean drug therapy costs increased but the mean chemotherapy costs decreased as the patient approached death (Fig 1C, Fig 1D, Fig 1E, S2 Table, S3 Table). For example, the mean inpatient drug therapy costs for AML more than doubled from $1,809 (SD, $2,204) in 12 months before death to $4,572 (SD, $4,923) in the last month; yet the mean chemotherapy costs decreased from $649 (SD, $890) to $530 (SD, $928) during the same period. The mean inpatient chemotherapy costs were consistently low for liver ($93 in 12 months before death to $77 in the last month) and thyroid cancers ($121 to $70, respectively); these costs were high initially but declined near the end of life for most of the other solid tumors. The mean inpatient drug therapy costs were markedly higher for AML than for other cancers. Among outpatients, the mean drug therapy and chemotherapy costs were high for kidney cancer in 12 months before death but dramatically declined near the end of life (from $929 to $369 for drug therapy and from $1,880 to $722 for chemotherapy, respectively); however, these costs remained stable for all other cancers throughout the year and declined slightly near death (Fig 2C, Fig 2D, Fig 2E, S2 Table, S3 Table).

## Discussion

This population-based study examined the monthly costs of EOL care per patient including drug therapy and chemotherapy for inpatient vs. outpatient care in the last year of life among decedents from one of the 13 most prevalent cancers in Korea based on national health insurance claims data. We showed that the monthly inpatient costs per patient increased as the patient approached death for all cancers while the monthly outpatient costs per patient decreased during the same period. Similarly, in the United States, inpatient costs per cancer decedent increased from $1,785 in 6 months before death to $20,559 in the last month, while outpatient costs decreased from $6,021 to $2,238, respectively [20].

Our data showed that the mean inpatient costs per patient increased steeply in the last few months of life for most cancers while the number of inpatients simultaneously increased. In addition, a substantial proportion of patients received chemotherapy in the inpatient setting in the last month of life, suggesting that a high proportion of EOL care costs incurred for inpatient care. This is consistent with the finding of another study in Korea; approximately 80% of spending per cancer decedent in Korea were associated with inpatient care [13]. These findings suggest that most cancer deaths in Korea occur in hospitals and that there are opportunities to reduce the costs and improve the quality of cancer care by providing greater access to hospice care at the end of life. In comparison, inpatient costs were responsible for 55% of total cancer-related costs in the last six months of life among the commercially insured U.S. patients; remaining 41% and 4% were for outpatient services and hospice costs, respectively [20].

While the proportion of outpatients receiving chemotherapy in the last month of life tended to be low, the ratio of the mean chemotherapy costs to the mean drug therapy costs was considerably high in these patients. This is probably because chemotherapy costs comprise most of drug therapy costs and not many other costs are incurred in the outpatient setting. On the contrary, the proportion of inpatients receiving chemotherapy tended to be high, but the ratio of the mean chemotherapy costs to the mean drug therapy costs was low among these patients. This is probably due to higher costs of non-pharmacological treatments used in the inpatient setting. These findings suggest the aggressiveness of cancer treatments even in the last months of life. Another study in Korea also documented a substantial proportion of cancer decedents receiving aggressive medical care near the end of life; 50.3% and 5.7% received chemotherapy in the last 2 months and 2 weeks of life, respectively [16]. Similarly, U.S. studies reported that older patients with cancer receive more aggressive EOL care including chemotherapy in a few weeks prior to death and that chemotherapy for patients with advanced cancer was associated with higher costs of EOL care [17,18].

We showed that the mean inpatient medical care costs of cancer per patient in the last year of life in Korea varied by cancer site with AML incurring the highest costs, which is consistent with the findings of a 2005 study [21]. We further showed that the inpatient costs for AML were more than twice the costs for other cancers and that the differences diverged even more in the last two months of life. As AML is a cancer that progresses fast, patients with AML often die while receiving chemotherapy. For that reason, the proportion of inpatients receiving chemotherapy in the last month of life may have been high (77%). The mean inpatient medical costs and drug therapy costs are markedly higher for AML than for other cancers; in contrast, the mean inpatient chemotherapy costs are comparable between AML and other cancers. This suggests that the costs of drug therapy other than chemotherapy per patient are substantial for AML; the costs may be attributable to blood transfusion, which is critical for the treatment of AML [22].

We showed that the mean outpatient chemotherapy costs for kidney cancer were very high initially and then gradually declined until the rate of decrease becomes steeper in the 3 months before death. It is plausible that recently introduced, orally administered, pricy targeted drugs might have been used for patients with kidney cancer in the last year of life until these therapies were discontinued when the patients approach the end of life [23,24]. Although only 27% of outpatients with kidney cancer received chemotherapy in the last month of life, the mean chemotherapy costs per patient were very high among these patients. Furthermore, the mean chemotherapy costs among outpatients with kidney cancer receiving chemotherapy in the last month of life were 1.96 times the mean drug therapy costs among outpatients receiving drug therapy, suggesting that highly expensive chemotherapy were used for patients with kidney cancer even in the last month of life.

The ratio of the mean chemotherapy costs to the mean drug therapy costs in the last month was highest for outpatients with thyroid cancer (1.98). This is probably because no other drugs than pricy chemotherapy were used to treat these patients [25]. Our findings suggest that only a small proportion (7%) of outpatients with thyroid cancer received pricy targeted chemotherapy in the last month of life. If this is the case, the mean drug therapy costs can be low among patients not receiving chemotherapy but the mean costs of targeted drug therapy can be high among the patients on chemotherapy.

Using national health insurance data, this study provides recent evidence on extraordinarily high costs of EOL cancer care in the inpatient setting that continue to increase until the last month of life. This analysis of population-based data presents system-wide costs of cancer care without a bias that can occur in an analysis of data from a limited number of patients or medical care institutions. Moreover, this is the first study to examine monthly cost trajectories for drug therapy and chemotherapy by cancer site and patient setting among end-stage cancer patients in Korea. We showed that the mean monthly inpatient care costs per cancer patient increased steeply very close to death for most cancers and that chemotherapy was used for a substantial proportion of patients even in the last month of life. These findings suggest the likelihood of unnecessary medical spending for terminal cancer care and highlight the importance of optimizing EOL care for cancer patients. Furthermore, our findings present policy implications for cancer care in Korea where hospice and home care services are not commonly used to care for terminal cancer patients [26]. While home care and hospice care services appropriate for terminal cancer patients are not widely available in Korea, the fee-for-service payment system in Korea provides an incentive for excessive medical care services. To control the unnecessarily aggressive and excessive treatment of cancer patients nearing death and resulting cost increases, policy interventions are needed, which could include a payment system reform (i.e., transition into capitation or value-based care) and promotion of health care providers’ communication with patients. In the U.S., having EOL discussions with the oncologist was suggested as a way to reduce medical care utilization and costs and to increase the quality of life of the patient [27]; the mean medical care costs in the last week of life were 35.7% lower among the patients who had EOL discussions, compared with $2,917 for those who did not [28].

Although this analysis of real world data provides a basis for developing evidence-based policy interventions for cancer care, this study has the following limitations. First, the HIRA database used in this study includes only medical care services that are reimbursed by the national health insurance system. Thus, non-reimbursable services such as caretaker services that were paid for by the patients out of pocket were not included in the analysis. Future studies should include costs of these services to estimate the entire economic burden of cancer care. Second, while being hospitalized for cancer, patients can receive inpatient care for unrelated conditions, suggesting the possibility of overestimation of cancer care costs. Conversely, if patients were primarily hospitalized for conditions other than cancer, cancer treatment costs may have been excluded from the analysis, thus underestimating cancer care costs. Third, our findings are descriptive only and do not account for differences in patient demographics or clinical profiles. The objective of this study was to describe cancer care costs in all cancer decedents in the last year of life by cancer site but not to investigate factors influencing the costs. The claim data used in our analysis were collected for the primary purpose of reimbursement. As a result, aside from patient sociodemographic information, the data include limited clinical information. An investigation of factors influencing cancer care costs would best be conducted by using claims data merged with an external data source with rich clinical information, such as electronic medical records, which should be left for future research.

## Conclusion

In conclusion, medical care costs for cancer in the last year of life showed different trends depending on patient setting. The mean monthly inpatient medical care costs per patient increased and the mean monthly outpatient costs per patient decreased for all cancers as the patient approached death. The mean monthly inpatient medical care costs were highest for AML and increased steeply in parallel with drug therapy over the last year of life. The mean monthly outpatient medical care, drug therapy, and chemotherapy costs were highest for kidney cancer and decreased sharply over the last year of life. A considerable proportion of patients received chemotherapy in the inpatient setting in the last month of life. Our findings highlight the need to examine the appropriateness of aggressive treatments including chemotherapy among patients with terminal cancer to reduce the undue financial burden on the patient, the family, and the insurance system.

## Supporting information

S1 Table Monthly inpatient and outpatient costs per patient with cancer in the last year of life. (DOCX)

S2 Table Mean drug therapy costs per patient in the last year of life by cancer site. (DOCX)

S3 Table Mean chemotherapy costs per patient in the last year of life by cancer site. (DOCX)
