# Supplementary material for: Medical care costs of cancer in the last year of life using national health insurance data in Korea
Source: PLoS One. 2018 Jun 7;13(6):e0197891. doi: 10.1371/journal.pone.0197891 (PMC5991689; doi:10.1371/journal.pone.0197891)
Supplement: S1 Table — (DOCX) [file pone.0197891.s001.docx]

**S1 Table. Monthly inpatient and outpatient costs per patient with cancer in the last year of life.**

| Cancer site | Month before death | 12 | | 11 | | 10 | | 9 | | 8 | | 7 | | 6 | | 5 | | 4 | | 3 | | 2 | | 1 | |  |
| --- | --- | --- | --- | --- | --- | --- | --- | --- | --- | --- | --- | --- | --- | --- | --- | --- | --- | --- | --- | --- | --- | --- | --- | --- | --- | --- |
|  |  | Mean costs ($) | SD | Mean costs ($) | SD | Mean costs ($) | SD | Mean costs ($) | SD | Mean costs ($) | SD | Mean costs ($) | SD | Mean costs ($) | SD | Mean costs ($) | SD | Mean costs ($) | SD | Mean costs ($) | SD | Mean costs ($) | SD | Mean costs ($) | SD | |
| AML | inpatient | 5,465 | 5,248 | 7,799 | 6,869 | 7,948 | 6,657 | 8,409 | 7,418 | 7,851 | 7,287 | 8,112 | 6,635 | 8,257 | 7,013 | 8,653 | 7,206 | 9,382 | 8,165 | 9,684 | 8,074 | 10,972 | 9,487 | 15,033 | 11,864 | |
|  | outpatient | 471 | 689 | 455 | 716 | 501 | 858 | 492 | 803 | 515 | 810 | 535 | 842 | 553 | 898 | 556 | 852 | 536 | 840 | 517 | 837 | 521 | 831 | 415 | 661 | |
| Stomach | inpatient | 1,771 | 1,692 | 1,988 | 1,785 | 2,006 | 1,775 | 2,036 | 1,922 | 2,042 | 1,897 | 2,093 | 1,902 | 2,130 | 1,900 | 2,232 | 2,099 | 2,320 | 2,210 | 2,504 | 2,244 | 2,872 | 2,629 | 3,791 | 3,691 | |
|  | outpatient | 336 | 459 | 327 | 462 | 320 | 448 | 324 | 445 | 321 | 449 | 313 | 421 | 302 | 428 | 309 | 446 | 307 | 439 | 295 | 422 | 282 | 406 | 241 | 348 | |
| Liver | inpatient | 2,472 | 3,546 | 2,580 | 2,819 | 2,571 | 3,009 | 2,606 | 3,251 | 2,610 | 3,134 | 2,591 | 2,715 | 2,656 | 2,961 | 2,715 | 3,030 | 2,755 | 3,061 | 2,863 | 3,509 | 3,156 | 3,799 | 4,303 | 5,252 | |
|  | outpatient | 489 | 770 | 466 | 736 | 487 | 773 | 491 | 805 | 504 | 819 | 513 | 828 | 506 | 828 | 504 | 821 | 495 | 818 | 460 | 763 | 394 | 646 | 286 | 455 | |
| Lung | inpatient | 1,869 | 1,807 | 2,132 | 2,080 | 2,160 | 2,071 | 2,169 | 2,073 | 2,177 | 2,055 | 2,224 | 2,082 | 2,275 | 2,088 | 2,366 | 2,205 | 2,483 | 2,304 | 2,629 | 2,455 | 2,954 | 2,742 | 3,889 | 3,806 | |
|  | outpatient | 598 | 890 | 571 | 860 | 571 | 866 | 559 | 842 | 558 | 860 | 520 | 812 | 534 | 856 | 521 | 826 | 511 | 825 | 485 | 801 | 437 | 717 | 322 | 530 | |
| Breast | inpatient | 1,571 | 1,498 | 1,729 | 1,426 | 1,809 | 1,607 | 1,835 | 1,643 | 1,926 | 1,778 | 1,870 | 1,657 | 2,043 | 1,997 | 2,123 | 2,029 | 2,290 | 2,057 | 2,547 | 2,217 | 2,897 | 2,494 | 3,953 | 3,596 | |
|  | outpatient | 600 | 816 | 591 | 837 | 568 | 769 | 554 | 723 | 557 | 788 | 571 | 785 | 560 | 783 | 552 | 755 | 542 | 810 | 520 | 765 | 437 | 652 | 341 | 518 | |
| Colorectal | inpatient | 1,882 | 1,735 | 2,134 | 1,920 | 2,074 | 1,831 | 2,110 | 1,937 | 2,123 | 1,917 | 2,203 | 2,151 | 2,230 | 2,087 | 2,277 | 2,152 | 2,461 | 2,366 | 2,553 | 2,633 | 2,837 | 2,916 | 3,626 | 4,060 | |
|  | outpatient | 359 | 568 | 350 | 553 | 364 | 594 | 354 | 569 | 354 | 565 | 343 | 555 | 339 | 554 | 338 | 527 | 333 | 547 | 323 | 523 | 302 | 472 | 247 | 367 | |
| Kidney | inpatient | 1,922 | 2,221 | 2,186 | 2,190 | 2,177 | 2,052 | 2,107 | 2,064 | 2,055 | 1,842 | 2,325 | 2,433 | 2,314 | 2,331 | 2,457 | 2,296 | 2,424 | 2,238 | 2,778 | 2,648 | 2,940 | 2,734 | 4,086 | 4,597 | |
|  | outpatient | 954 | 1,346 | 1,014 | 1,398 | 935 | 1,387 | 895 | 1,252 | 926 | 1,283 | 903 | 1,262 | 831 | 1,248 | 794 | 1,166 | 771 | 1,131 | 700 | 1,092 | 583 | 1,031 | 424 | 736 | |
| Prostate | inpatient | 1,288 | 1,173 | 1,644 | 1,317 | 1,716 | 1,315 | 1,709 | 1,478 | 1,817 | 1,600 | 1,766 | 1,536 | 1,822 | 1,495 | 1,969 | 1,908 | 1,947 | 1,846 | 2,129 | 2,009 | 2,356 | 2,458 | 3,008 | 3,478 | |
|  | outpatient | 357 | 769 | 373 | 743 | 362 | 682 | 359 | 700 | 364 | 733 | 358 | 681 | 355 | 670 | 362 | 688 | 350 | 641 | 373 | 705 | 351 | 676 | 294 | 520 | |
| Non-Hodgkin’s lymphoma | inpatient | 2,209 | 2,713 | 2,920 | 3,945 | 3,347 | 4,341 | 3,545 | 4,390 | 3,236 | 3,231 | 3,385 | 3,222 | 3,560 | 4,069 | 3,802 | 4,149 | 3,875 | 4,096 | 4,232 | 5,201 | 4,514 | 5,147 | 6,630 | 8,213 | |
|  | outpatient | 356 | 594 | 298 | 507 | 295 | 463 | 335 | 683 | 328 | 516 | 367 | 647 | 372 | 695 | 338 | 606 | 358 | 646 | 336 | 665 | 340 | 716 | 292 | 709 | |
| Cervical | inpatient | 1,760 | 1,490 | 2,164 | 2,056 | 2,177 | 2,086 | 2,185 | 1,955 | 2,278 | 1,968 | 2,394 | 2,190 | 2,402 | 2,236 | 2,761 | 2,590 | 2,909 | 2,627 | 3,269 | 3,153 | 3,623 | 3,138 | 4,294 | 3,851 | |
|  | outpatient | 596 | 1,013 | 580 | 1,003 | 549 | 1,041 | 498 | 861 | 488 | 868 | 518 | 956 | 482 | 871 | 385 | 636 | 389 | 647 | 363 | 681 | 317 | 559 | 259 | 449 | |
| Ovarian | inpatient | 1,746 | 1,446 | 2,130 | 1,996 | 2,211 | 2,155 | 2,108 | 2,061 | 2,164 | 1,907 | 2,180 | 2,021 | 2,315 | 2,087 | 2,537 | 2,397 | 2,796 | 2,607 | 3,056 | 2,900 | 3,590 | 3,218 | 4,603 | 4,344 | |
|  | outpatient | 298 | 414 | 298 | 426 | 295 | 420 | 313 | 436 | 304 | 487 | 306 | 462 | 306 | 465 | 307 | 477 | 296 | 449 | 286 | 529 | 283 | 469 | 247 | 366 | |
| Pancreas | inpatient | 2,306 | 2,369 | 2,596 | 2,608 | 2,398 | 2,425 | 2,447 | 2,288 | 2,455 | 2,427 | 2,385 | 2,404 | 2,380 | 2,273 | 2,466 | 2,311 | 2,533 | 2,313 | 2,670 | 2,297 | 2,979 | 2,474 | 3,860 | 3,211 | |
|  | outpatient | 583 | 809 | 542 | 739 | 548 | 740 | 557 | 763 | 544 | 763 | 532 | 709 | 505 | 711 | 476 | 664 | 448 | 647 | 405 | 591 | 351 | 492 | 277 | 393 | |
| Thyroid | inpatient | 1,329 | 1,261 | 1,897 | 1,397 | 1,759 | 1,454 | 1,830 | 1,507 | 2,042 | 1,736 | 2,286 | 2,347 | 2,217 | 2,219 | 2,536 | 2,391 | 2,316 | 2,047 | 2,427 | 2,361 | 2,914 | 2,963 | 3,494 | 3,973 | |
|  | outpatient | 282 | 718 | 265 | 699 | 231 | 667 | 268 | 762 | 261 | 585 | 309 | 841 | 363 | 840 | 341 | 823 | 321 | 723 | 352 | 838 | 334 | 820 | 248 | 470 | |

Costs in Korean won were converted to US$ using the conversion rate of 1,100 won/US$.
