# Supplementary material for: Medical care costs of cancer in the last year of life using national health insurance data in Korea
Source: PLoS One. 2018 Jun 7;13(6):e0197891. doi: 10.1371/journal.pone.0197891 (PMC5991689; doi:10.1371/journal.pone.0197891)
Supplement: S2 Table — (DOCX) [file pone.0197891.s002.docx]

**S2 Table. Mean drug therapy costs per patient in the last year of life by cancer site.**

| Cancer site | Month before death | 12 | | 11 | | 10 | | 9 | | 8 | | 7 | | 6 | | 5 | | 4 | | 3 | | 2 | | 1 | |
| --- | --- | --- | --- | --- | --- | --- | --- | --- | --- | --- | --- | --- | --- | --- | --- | --- | --- | --- | --- | --- | --- | --- | --- | --- | --- |
|  | Setting | Amount ($) | SD | Amount ($) | SD | Amount ($) | SD | Amount ($) | SD | Amount ($) | SD | Amount ($) | SD | Amount ($) | SD | Amount ($) | SD | Amount ($) | SD | Amount ($) | SD | Amount ($) | SD | Amount ($) | SD |
| AML | inpatient | 1,809 | 2,204 | 2,772 | 3,113 | 2,782 | 3,086 | 2,995 | 3,420 | 2,804 | 3,290 | 2,904 | 3,289 | 2,881 | 3,104 | 3,051 | 3,311 | 3,239 | 3,687 | 3,304 | 3,657 | 3,690 | 4,257 | 4,572 | 4,923 |
|  | outpatient | 322 | 620 | 330 | 657 | 355 | 852 | 349 | 735 | 347 | 685 | 355 | 691 | 354 | 689 | 339 | 644 | 282 | 579 | 278 | 582 | 245 | 533 | 194 | 461 |
| Stomach | inpatient | 676 | 615 | 698 | 611 | 696 | 615 | 698 | 633 | 692 | 626 | 703 | 637 | 692 | 636 | 695 | 665 | 685 | 690 | 694 | 715 | 763 | 838 | 950 | 1,093 |
|  | outpatient | 199 | 340 | 199 | 356 | 200 | 361 | 197 | 338 | 184 | 298 | 182 | 303 | 180 | 322 | 177 | 319 | 171 | 297 | 163 | 269 | 153 | 235 | 132 | 220 |
| Liver | inpatient | 486 | 1,573 | 478 | 1,111 | 476 | 1,121 | 491 | 1,292 | 500 | 1,286 | 481 | 934 | 506 | 1,032 | 525 | 1,039 | 553 | 1,000 | 595 | 1,930 | 683 | 1,803 | 940 | 1,482 |
|  | outpatient | 285 | 601 | 277 | 594 | 283 | 607 | 296 | 645 | 296 | 625 | 309 | 649 | 303 | 652 | 301 | 628 | 313 | 665 | 288 | 589 | 244 | 518 | 165 | 356 |
| Lung | inpatient | 642 | 724 | 650 | 733 | 656 | 728 | 667 | 757 | 652 | 713 | 655 | 729 | 666 | 754 | 665 | 754 | 661 | 738 | 665 | 746 | 709 | 777 | 919 | 1,006 |
|  | outpatient | 387 | 705 | 370 | 675 | 369 | 668 | 351 | 644 | 342 | 631 | 321 | 598 | 314 | 589 | 302 | 566 | 290 | 553 | 270 | 513 | 253 | 462 | 201 | 378 |
| Breast | inpatient | 670 | 736 | 665 | 677 | 648 | 691 | 650 | 711 | 643 | 675 | 611 | 644 | 613 | 662 | 610 | 657 | 617 | 664 | 623 | 724 | 679 | 780 | 864 | 1,005 |
|  | outpatient | 387 | 603 | 370 | 594 | 358 | 569 | 362 | 560 | 362 | 597 | 372 | 601 | 349 | 572 | 339 | 555 | 310 | 514 | 304 | 514 | 248 | 421 | 179 | 336 |
| Colorectal | inpatient | 787 | 777 | 777 | 806 | 776 | 819 | 736 | 820 | 719 | 790 | 719 | 823 | 706 | 822 | 670 | 796 | 670 | 774 | 662 | 815 | 719 | 922 | 909 | 1,295 |
|  | outpatient | 208 | 396 | 209 | 393 | 200 | 393 | 197 | 378 | 199 | 363 | 186 | 354 | 190 | 361 | 183 | 322 | 180 | 322 | 170 | 289 | 166 | 275 | 137 | 225 |
| Kidney | inpatient | 445 | 635 | 449 | 617 | 520 | 751 | 493 | 666 | 505 | 772 | 509 | 793 | 491 | 699 | 568 | 846 | 576 | 860 | 679 | 952 | 710 | 914 | 948 | 1,312 |
|  | outpatient | 929 | 1,393 | 1,018 | 1,416 | 966 | 1,471 | 902 | 1,296 | 910 | 1,352 | 862 | 1,271 | 776 | 1,227 | 734 | 1,171 | 687 | 1,119 | 609 | 1,033 | 496 | 929 | 369 | 762 |
| Prostate | inpatient | 368 | 462 | 360 | 460 | 361 | 460 | 354 | 429 | 366 | 475 | 354 | 432 | 355 | 456 | 376 | 503 | 384 | 537 | 438 | 575 | 497 | 661 | 662 | 916 |
|  | outpatient | 241 | 450 | 257 | 486 | 259 | 506 | 265 | 568 | 267 | 532 | 260 | 507 | 268 | 534 | 277 | 590 | 265 | 522 | 272 | 567 | 266 | 557 | 209 | 390 |
| Non-Hodgkin’s lymphoma | inpatient | 877 | 1,485 | 1,060 | 2,131 | 1,081 | 1,950 | 1,215 | 2,310 | 1,019 | 1,527 | 958 | 1,143 | 1,068 | 1,703 | 1,067 | 1,644 | 1,089 | 1,511 | 1,241 | 1,960 | 1,190 | 1,757 | 1,610 | 2,629 |
|  | outpatient | 143 | 192 | 143 | 220 | 144 | 254 | 131 | 199 | 175 | 313 | 162 | 266 | 158 | 241 | 136 | 231 | 137 | 207 | 140 | 232 | 141 | 244 | 109 | 198 |
| Cervical | inpatient | 506 | 455 | 539 | 500 | 543 | 501 | 575 | 529 | 596 | 531 | 624 | 571 | 624 | 643 | 680 | 685 | 713 | 815 | 775 | 937 | 889 | 996 | 1,036 | 1,198 |
|  | outpatient | 142 | 161 | 131 | 177 | 136 | 170 | 138 | 183 | 137 | 184 | 138 | 200 | 137 | 201 | 132 | 172 | 136 | 193 | 140 | 199 | 147 | 269 | 128 | 271 |
| Ovarian | inpatient | 747 | 575 | 794 | 669 | 793 | 669 | 776 | 682 | 774 | 679 | 753 | 664 | 759 | 670 | 764 | 718 | 792 | 776 | 829 | 848 | 907 | 918 | 1,093 | 1,180 |
|  | outpatient | 161 | 239 | 166 | 277 | 174 | 288 | 171 | 274 | 160 | 281 | 158 | 266 | 170 | 329 | 157 | 289 | 152 | 274 | 129 | 242 | 126 | 176 | 135 | 237 |
| Pancreas | inpatient | 558 | 598 | 597 | 640 | 572 | 614 | 578 | 600 | 581 | 618 | 581 | 639 | 576 | 617 | 589 | 643 | 604 | 673 | 641 | 712 | 735 | 781 | 956 | 1,008 |
|  | outpatient | 368 | 579 | 373 | 571 | 373 | 581 | 381 | 593 | 380 | 624 | 370 | 566 | 351 | 545 | 333 | 532 | 309 | 497 | 272 | 446 | 239 | 389 | 177 | 279 |
| Thyroid | inpatient | 218 | 308 | 266 | 414 | 222 | 235 | 292 | 452 | 254 | 286 | 334 | 470 | 359 | 534 | 391 | 498 | 346 | 430 | 440 | 555 | 602 | 716 | 751 | 924 |
|  | outpatient | 103 | 207 | 122 | 323 | 109 | 326 | 124 | 398 | 127 | 299 | 156 | 549 | 143 | 345 | 156 | 419 | 158 | 414 | 166 | 391 | 165 | 419 | 129 | 258 |

Costs in Korean won were converted to US$ using the conversion rate of 1,100 won/US$.
