# Supplementary material for: Medical care costs of cancer in the last year of life using national health insurance data in Korea
Source: PLoS One. 2018 Jun 7;13(6):e0197891. doi: 10.1371/journal.pone.0197891 (PMC5991689; doi:10.1371/journal.pone.0197891)
Supplement: S3 Table — (DOCX) [file pone.0197891.s003.docx]

**S3 Table. Mean chemotherapy costs per patient in the last year of life by cancer site.**

| Cancer site | Month before death | 12 | | 11 | | 10 | | 9 | | 8 | | 7 | | 6 | | 5 | | 4 | | 3 | | 2 | | 1 | |
| --- | --- | --- | --- | --- | --- | --- | --- | --- | --- | --- | --- | --- | --- | --- | --- | --- | --- | --- | --- | --- | --- | --- | --- | --- | --- |
|  | Setting | Amount ($) | SD | Amount ($) | SD | Amount ($) | SD | Amount ($) | SD | Amount ($) | SD | Amount ($) | SD | Amount ($) | SD | Amount ($) | SD | Amount ($) | SD | Amount ($) | SD | Amount ($) | SD | Amount ($) | SD |
| AML | inpatient | 649 | 890 | 631 | 880 | 600 | 781 | 608 | 840 | 546 | 715 | 570 | 824 | 640 | 889 | 620 | 913 | 547 | 825 | 489 | 781 | 461 | 808 | 530 | 928 |
|  | outpatient | 617 | 1,109 | 624 | 1,106 | 598 | 1,492 | 710 | 1,183 | 602 | 1,055 | 495 | 1,024 | 484 | 931 | 374 | 838 | 310 | 784 | 305 | 808 | 206 | 617 | 170 | 525 |
| Stomach | inpatient | 593 | 551 | 587 | 528 | 570 | 520 | 558 | 515 | 535 | 488 | 524 | 494 | 493 | 479 | 457 | 469 | 403 | 450 | 322 | 402 | 218 | 345 | 126 | 302 |
|  | outpatient | 390 | 486 | 386 | 520 | 377 | 498 | 373 | 486 | 337 | 412 | 322 | 424 | 314 | 458 | 287 | 450 | 253 | 401 | 215 | 350 | 167 | 276 | 111 | 237 |
| Liver | inpatient | 93 | 253 | 95 | 282 | 97 | 267 | 97 | 270 | 102 | 300 | 106 | 296 | 113 | 320 | 113 | 321 | 123 | 333 | 117 | 333 | 95 | 291 | 77 | 261 |
|  | outpatient | 626 | 1,034 | 586 | 1,017 | 617 | 1,024 | 638 | 1,047 | 604 | 992 | 624 | 1,013 | 602 | 1,022 | 561 | 959 | 578 | 990 | 470 | 850 | 361 | 756 | 196 | 518 |
| Lung | inpatient | 547 | 732 | 518 | 719 | 519 | 697 | 511 | 713 | 495 | 683 | 484 | 683 | 478 | 696 | 446 | 674 | 392 | 618 | 332 | 573 | 239 | 468 | 162 | 396 |
|  | outpatient | 779 | 957 | 727 | 918 | 696 | 900 | 655 | 876 | 616 | 853 | 562 | 815 | 516 | 795 | 474 | 767 | 435 | 748 | 369 | 689 | 313 | 589 | 234 | 464 |
| Breast | inpatient | 623 | 721 | 588 | 656 | 554 | 632 | 545 | 633 | 509 | 562 | 469 | 544 | 440 | 502 | 398 | 471 | 372 | 485 | 316 | 498 | 233 | 391 | 153 | 344 |
|  | outpatient | 557 | 730 | 523 | 713 | 498 | 696 | 509 | 674 | 511 | 725 | 518 | 727 | 486 | 701 | 462 | 672 | 415 | 642 | 406 | 644 | 321 | 534 | 228 | 472 |
| Colorectal | inpatient | 704 | 743 | 683 | 781 | 676 | 787 | 633 | 780 | 602 | 760 | 580 | 760 | 539 | 759 | 474 | 712 | 402 | 647 | 307 | 569 | 207 | 489 | 140 | 445 |
|  | outpatient | 392 | 603 | 371 | 574 | 343 | 589 | 337 | 548 | 325 | 527 | 298 | 524 | 283 | 520 | 245 | 440 | 214 | 393 | 168 | 340 | 133 | 290 | 96 | 220 |
| Kidney | inpatient | 370 | 713 | 334 | 633 | 460 | 819 | 375 | 665 | 402 | 854 | 346 | 805 | 297 | 617 | 415 | 861 | 407 | 885 | 412 | 877 | 297 | 717 | 212 | 553 |
|  | outpatient | 1,880 | 1,546 | 1,929 | 1,517 | 1,901 | 1,644 | 1,766 | 1,418 | 1,752 | 1,494 | 1,715 | 1,397 | 1,548 | 1,430 | 1,440 | 1,390 | 1,285 | 1,352 | 1,152 | 1,279 | 912 | 1,225 | 722 | 1,085 |
| Prostate | inpatient | 400 | 437 | 348 | 437 | 338 | 452 | 310 | 388 | 301 | 459 | 258 | 345 | 236 | 380 | 213 | 337 | 201 | 374 | 186 | 397 | 167 | 395 | 153 | 376 |
|  | outpatient | 303 | 561 | 317 | 603 | 325 | 636 | 338 | 715 | 336 | 663 | 325 | 638 | 329 | 663 | 352 | 740 | 321 | 640 | 324 | 700 | 324 | 700 | 255 | 500 |
| Non-Hodgkin’s lymphoma | inpatient | 683 | 1,146 | 693 | 1,777 | 544 | 1,104 | 492 | 1,347 | 389 | 675 | 340 | 532 | 360 | 641 | 378 | 929 | 357 | 701 | 376 | 787 | 269 | 615 | 371 | 1,603 |
|  | outpatient | 106 | 261 | 71 | 82 | 41 | 59 | 49 | 55 | 134 | 294 | 161 | 397 | 107 | 242 | 99 | 256 | 80 | 182 | 102 | 252 | 105 | 265 | 55 | 152 |
| Cervical | inpatient | 379 | 384 | 366 | 384 | 360 | 383 | 361 | 382 | 344 | 369 | 341 | 369 | 315 | 425 | 295 | 387 | 271 | 408 | 208 | 371 | 139 | 269 | 115 | 308 |
|  | outpatient | 153 | 188 | 141 | 170 | 167 | 194 | 162 | 206 | 163 | 270 | 120 | 184 | 109 | 173 | 113 | 165 | 113 | 162 | 110 | 162 | 76 | 79 | 65 | 69 |
| Ovarian | inpatient | 631 | 507 | 636 | 548 | 642 | 557 | 621 | 580 | 589 | 555 | 567 | 539 | 528 | 520 | 492 | 533 | 453 | 533 | 375 | 498 | 263 | 428 | 214 | 455 |
|  | outpatient | 330 | 386 | 405 | 501 | 419 | 477 | 357 | 446 | 365 | 497 | 368 | 484 | 366 | 581 | 312 | 531 | 289 | 482 | 186 | 377 | 103 | 172 | 160 | 393 |
| Pancreas | inpatient | 278 | 527 | 267 | 492 | 261 | 491 | 256 | 480 | 263 | 497 | 262 | 509 | 257 | 497 | 225 | 449 | 200 | 424 | 169 | 382 | 121 | 304 | 74 | 210 |
|  | outpatient | 578 | 698 | 570 | 684 | 573 | 695 | 589 | 699 | 558 | 746 | 523 | 647 | 496 | 633 | 454 | 619 | 390 | 572 | 323 | 512 | 223 | 406 | 141 | 297 |
| Thyroid | inpatient | 121 | 332 | 35 | 49 | 40 | 84 | 37 | 57 | 39 | 54 | 28 | 42 | 55 | 138 | 54 | 148 | 59 | 159 | 51 | 126 | 79 | 243 | 70 | 223 |
|  | outpatient | 232 | 547 | 415 | 812 | 391 | 914 | 491 | 1,118 | 333 | 686 | 735 | 1,517 | 419 | 799 | 515 | 927 | 495 | 974 | 467 | 858 | 452 | 858 | 255 | 513 |

Costs in Korean won were converted to US$ using the conversion rate of 1,100 won/US$.
